# Male sea lamprey countersignal relative to their baseline pheromone but not the intensity of rivals’ signals

**DOI:** 10.1098/rsbl.2025.0108

**Published:** 2025-07-09

**Authors:** Tyler J. Buchinger, Skye D. Fissette, Ugo Bussy, Belinda Huerta, Sonam Tamrakar, Weiming Li

**Affiliations:** ^1^ Department of Fisheries and Wildlife, Michigan State University, East Lansing, MI, USA

**Keywords:** chemical communication, eavesdropping, competitive risk, rival assessment

## Abstract

Animals signalling to potential mates inadvertently reveal information to sexual rivals. In species that communicate with visual or acoustic signals, rivals are well documented to use this information to optimize their own signalling strategy based on the current level of competitive risk. We studied how males fine-tune their signals after exposure to varying levels of simulated competition in a species that relies on chemical signals, the sea lamprey (*Petromyzon marinus*). Sea lamprey aggregate on spawning grounds in streams, where males each build a nest and signal to females using the sex pheromone 3-keto petromyzonol sulphate (3kPZS). We hypothesized that males use the concentration of environmental 3kPZS to infer the level of competitive risk and adjust their 3kPZS release proportionally. Males increased 3kPZS release after exposure to 3kPZS but, contrary to our hypothesis, the change in release was similar across concentrations from 5 × 10^−7^ M down to 5 × 10^−13^ M. Interestingly, the increase in 3kPZS release after exposure to 3kPZS was negatively correlated with baseline release rates. Taken together, our results indicate male sea lamprey adjust their pheromone signals based on the presence of rivals and their own baseline signal but not any graded assessment of competition risk.

## 1. Introduction

Males seeking potential mates often eavesdrop on sexual advertisements of rivals [1]. Doing so allows males to capitalize on rivals’ efforts to attract or detect females [2] or optimize their signalling strategy given the current competitive risk [3–5]. The benefits of eavesdropping on rivals, together with selection for male–male communication, have led males in some species to detect and discern among signals of sexual rivals with sensitivity comparable to females’ refined ‘taste for the beautiful’ [6]. For example, male túngara frogs (*Engystomops pustulosus*) adjust their calls based on the complexity [7] and frequency [8] of rivals’ calls to females, as well as the ripples produced when rivals vocalize [9]. Such vigilance towards rivals’ signals to females is well documented for acoustic and visual communication [10–13], but not for chemical communication (but see [14]).

Sea lamprey (*Petromyzon marinus*) provide a rare example of males eavesdropping on chemical signals of sexual rivals [15]. In this primarily nocturnal jawless fish, males construct spawning nests on gravel bars in streams and defend them against intruding males [16,17]. Females navigate upstream to a nest following a male pheromone whose major component is 3-keto petromyzonol sulphate (3kPZS), a bile acid produced in the liver and released through the gills [18,19]. Release rates of 3kPZS vary within and among males [20,21] and appear to be under sexual selection [22] due to female preference for higher pheromone concentrations [15,23]. Although the male pheromone primarily influences female behaviour [24], males can also detect 3kPZS [25] and are likely to encounter pheromones from competing males given they spawn in lek-like aggregations [16,17] and continuously release 3kPZS once sexually mature [21]. Males are unlikely to frequently auto-detect their own pheromones, as sea lamprey spawn in flowing water and a male’s pheromone will usually disperse downstream of his nose. As in species that communicate with visual and acoustic signals, male sea lamprey appear to use information gleaned from competitors’ signals to optimize their own signalling based on the presence of competitors; males experimentally exposed to 3kPZS increase their release of it and are more attractive to females than males not exposed to 3kPZS [15].

Can male sea lamprey gauge the competition by smelling rivals as keenly as males in species that watch and listen to rivals? In our previous study, males adjusted their pheromone release after exposure to 5 × 10^–10^ M 3kPZS [15]. Females, however, can detect male pheromone molecules at much lower concentrations (<1 × 10^−14^ M) [25] and select odorants with even slightly higher 3kPZS concentrations (<2× difference) [15,23]. As males show similar pheromone sensitivity as females [26,27], they could conceivably assess the concentration of 3kPZS and make measured changes to their signals given the level of competitive risk. However, males do not track 3kPZS plumes to find the source but rather get whiffs of rivals while on their own nest or traversing spawning grounds. Whether these whiffs can capture precise information about the level of competitive risk seems uncertain; unlike light and sound, chemicals such as 3kPZS move via diffusion or flow and therefore disperse into patchy spatial distributions whose concentrations depend on fluid currents and volume [28,29]. A second source of ambiguity in the information associated with 3kPZS concentrations is the males themselves; 3kPZS release rates are highly variable within and among males [20,21]. Plume dynamics, variation in stream discharge and variation in 3kPZS release rates may degrade any links between the concentration a male detects and the number and location of rivals.

In the current study, we tested the hypothesis that male sea lamprey respond to rivals’ pheromones with graded adjustments to their own signals that are proportionate to the perceived level of competition. Specifically, we determined the change in 3kPZS release rate after exposure to synthesized 3kPZS at different concentrations ranging from supranormal (5 × 10^−7^ M) to below their detection threshold (5 × 10^−15^ M). As males might also respond to competitors based on their own internal state (i.e. ‘self-assessment’) [30], we leveraged our relatively large set of observations to also investigate the effects of males’ baseline release rates on the magnitude of their responses to a simulated competitor.

## 2. Methods

### (a) Experimental animals

Migratory sea lamprey were trapped in tributaries of Lakes Michigan and Huron by the US Fish and Wildlife Service and Fisheries and Oceans Canada. Captured lamprey were transported to the US Geological Survey Hammond Bay Biological Station (HBBS), Millersburg, MI, USA, and held in 200−1000 l tanks that were supplied with aerated, ambient temperature Lake Huron water. Later, groups of immature males were held in cages (0.25−1 m^3^, approx. 25 individuals per cage) in the Ocqueoc River, Millersburg, MI, until they became sexually mature. Sexual maturation was assessed daily by applying gentle abdominal pressure [31] and males with expressible milt (sexually mature) were transported back to HBBS and held as groups in 200 l flow-through tanks until experimental use (<48 h). Only sexually mature males were used in experiments.

### (b) Experimental design

We measured the release of natural 3kPZS by males before and after they were exposed to synthesized 3kPZS using established methods [15]. Importantly, measuring pheromone release required us to hold males in static water, which is critical to allow released pheromone to accumulate in the water but also creates the ecologically rare scenario of males smelling themselves. To begin, individual males were placed in 5 gallon (approx. 19 l) buckets. Each bucket had a hole in the bottom that was plugged with a rubber stopper and a drain in the side that limited the maximum water volume in the bucket to 5 l. Males were acclimated for 1 h, during which buckets were supplied with aerated, ambient temperature Lake Huron water. After acclimation, the water supply was shut off and the water in the bucket drained by removing the rubber plug without removing the lamprey (to avoid stress of physical capture). Each bucket was then (within <1 min) filled with 3 l of deionized water (Series 1) or Lake Huron water (Series 2 and 3). Water temperatures in buckets were 17.5 ± 1.2°C during the acclimation period and 17.2 ± 1.0°C at the end of the exposure period (mean ± s.d.; difference = 0–3.1°C, median 0.5°C). Lake Huron water was used during *Series 2* and *3* because deionized water was unavailable during experiments (note Lake Huron has generally low ion concentrations) [32]. Lake Huron water had low concentrations of pheromone (3kPZS 0.006 ± 0.004 ng ml^−1^, mean ± s.d.; *n* = 12). Thirty minutes later, 50 ml of water was sampled from each bucket to establish each male’s baseline rate of 3kPZS release prior to pheromone treatment. Each sample was immediately spiked with an internal standard of 5-deuterated 3kPZS ([^2^H_5_] 3kPZS, 5d-3kPZS; Bridge Organics Inc., Vicksburg, MI, USA) to reach a concentration of 0.1 ng ml^−1^. After collecting baseline samples, males were treated with 5d-3kPZS (see below) or a vehicle control (1 : 1 methanol : water) by pouring 1 ml of each solution in the bucket and gently mixing the water. Males were sampled in sets of *n* = 8, with each male receiving a randomly selected treatment. Post-treatment release rates were assessed 10 min after exposure using water sampled as described above, except 5d-3kPZS treatment samples were spiked with 11-deuterated 3kPZS (11d-3kPZS; Bridge Organics Inc.), which allowed us to discern the internal standard from the treatment (5d-3kPZS). Finally, 10 ml sub-samples were decanted from each 50 ml sample and all samples were frozen at −20°C. The concentration of 3kPZS in each 10 ml sample was quantified using ultra high-performance liquid chromatography tandem mass spectrometry as previously described [15,33].

Three series of experiments determined 3kPZS release by males exposed to 5d-3kPZS across a range of concentrations. Treating males with 5d-3kPZS allowed us to differentiate between 3kPZS released by males and 3kPZS that was experimentally added. Repeated series (one per year: 2016, 2017 and 2021) that included decreasingly lower concentrations were needed to identify the threshold at which males cease to respond to a simulated competitor. All series tested concentrations that differed by one order of magnitude. Series 1: 5 × 10^–7^ M to 5 × 10^–13^ M; Series 2: 5 × 10^–12^ M to 5 × 10^–14^ M; Series 3: 5 × 10^–12^ M to 5 × 10^–16^ M.

### (c) Statistical analysis

Rates of 3kPZS release (mg h^−1^) were calculated using concentrations measured in water (ng ml^−1^) and compared between time points (baseline and 10 min) and across treatments [15]. Data from baseline and 10 min samples were discarded if the internal standard was not detected, the 3kPZS concentration did not increase between timepoints (male not observed to release 3kPZS), or in a few cases when samples were lost [15]. In Series 3, a large number of males released very little 3kPZS, possibly due to issues in evaluation of sexual maturity (immature males release little 3kPZS) [18,34]. Therefore, males were also excluded (*n* = 1 from Series 1, *n* = 24 from Series 3) if they did not release more 3kPZS than the maximum release rate calculated for Lake Huron water that was sampled as a control but not conditioned with males (0.00025 mg h^−1^; *n* = 12). After removing males with low release rates, the 2021 control treatment had *n* = 5 and 5 × 10^–16^ M treatment had *n* = 4 whereas all other treatments had 9−13 replicates. All statistical analyses were performed using R v. 4.3.1 [35].

We first analysed the effect of pheromone exposure on 3kPZS release rates using non-parametric longitudinal analysis (F1-LD-F1 models) in the nparLD package [36]. Separate models for each series included a whole-plot factor (treatment) and one sub-plot factor (time). A significant treatment × time interaction would indicate pheromone treatments have different effects on the change in release rates between the baseline and 10 min sampling points. The *f1.ld.f1* function was used for post hoc pairwise comparisons between treatments, with *p*-values adjusted using a Benjamini & Hochberg (BH) correction. For Series 3, the vehicle control and 10^−16^ M treatments were dropped due to low sample sizes after removing males that released little 3kPZS (see above). In this case, baseline release rates were used as a substitute for the vehicle control to determine whether exposure to each pheromone treatment resulted in increased 3kPZS release. Note that Fissette *et al.* [15] and our results from Series 1 and Series 2 indicate baseline release rates ≈10 min release rates for males not exposed to 5d-3kPZS. For Series 3, baseline release rates and post-treatment release rates were first compared using Wilcoxon signed-rank tests and then treatments that resulted in increased pheromone release were tested using an F1-LD-F1 model.

Second, we investigated the effects of males’ baseline release rates on the magnitude of their responses to a simulated competitor. For this analysis, we combined data from all treatments for which we observed a significant increase in 3kPZS release after exposure to 5d-3kPZS, including data previously published by Fissette *et al.* [15]. The datasets were combined to maximize our statistical power. We then evaluated whether the change in release rate between the baseline (*x*) and the 10 min sample (*y*) differed according to the baseline release rate. As correlating a fractional increase of *x* with *x* itself could generate spurious results [37], we used the correction suggested by Tu [38]. Specifically, we correlated *x* with y/x and compared $r_{x,\;y/x}$ to the expected ‘null correlation’ of *r*_null_ = $r_{x,y\;}-\;1/\sqrt{2\left(1\;-\;r_{x,\;\;y}\right)}$. The observed correlation $r_{x,\;y/x}$ and null correlation *r*_null_ are then each *z*-transformed where *z_r_* (*r*) = 1/2ln⁡(1 + r/1 − r) and compared using a *z*-test where *z* = $z_{r}\left(r_{x,\;\;y/x}\right)\;-\;z_{r}\left(r_{null}\right)\;/\sqrt{\frac{1}{\left(n-3\right)}}$.

## 3. Results

**Figure 1.**
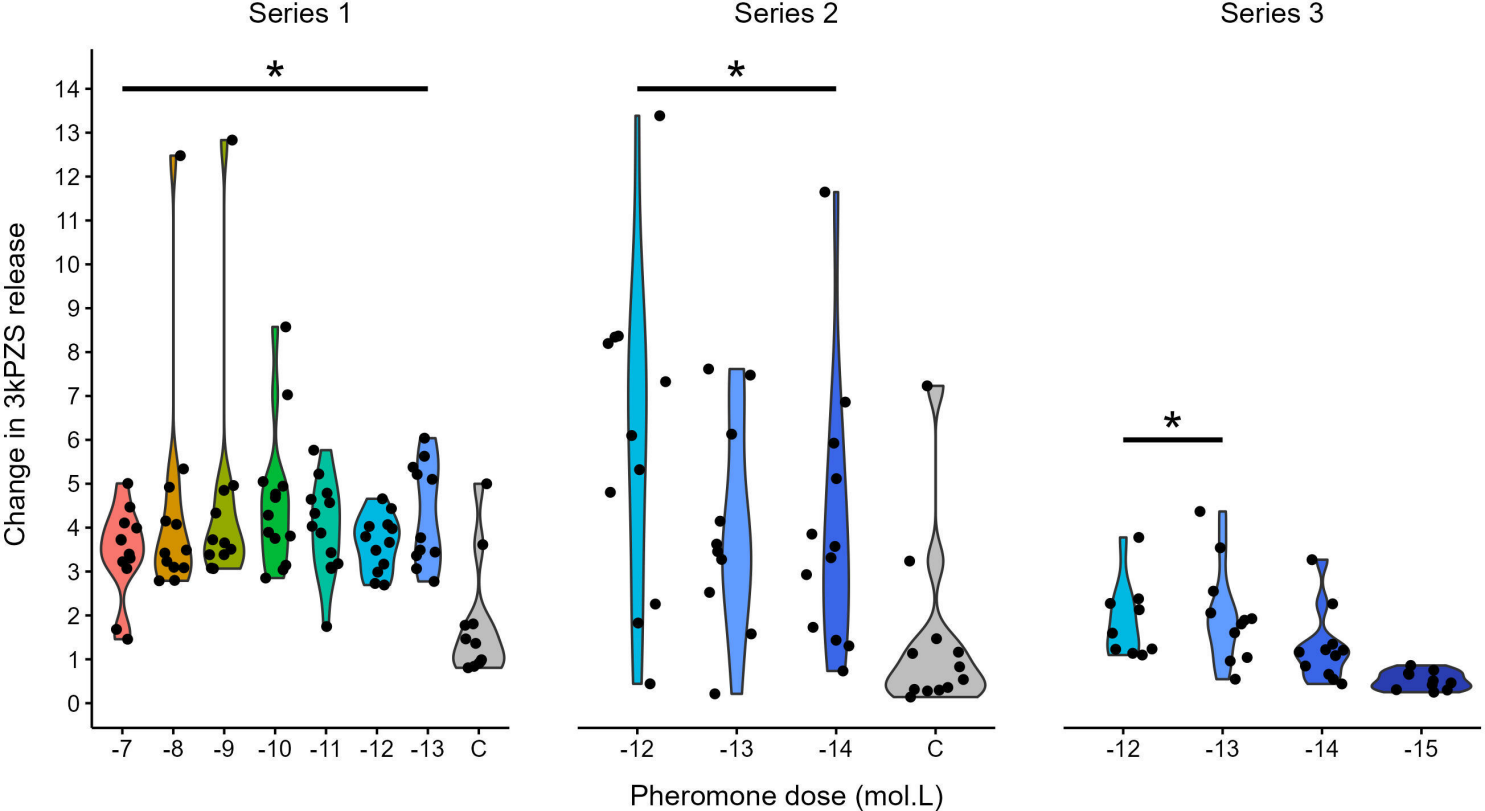
Magnitude of the competing signal does not affect the increase in pheromone release by male sea lamprey (*Petromyzon marinus*) exposed to simulated competition. Release rates (mg h^−1^) of the pheromone 3-keto petromyzonol sulfate (3kPZS) were determined before and 10 min after exposure to 5-deuterated 3kPZS ([^2^H_5_] 3kPZS, 5d-3kPZS). Change in 3kPZS release after exposure to 3kPZS was calculated as the end release rate after exposure divided by the baseline release rate before exposure. Asterisks indicate significantly higher 3kPZS release rates after exposure to 5d-3kPZS when compared to a vehicle control treatment (C) (Series 1 and Series 2; non-parametric longitudinal analysis with Benjamini & Hochberg correction for multiple comparisons) or pre-exposure release rates (Series 3; Wilcoxon signed-rank tests). Data for the control treatment were unavailable for Series 3 (see text). Within each series, treatment groups under the bar were not significantly different. *n* = 9–13 for each treatment.

Male sea lamprey increased pheromone release after exposure to 5d-3kPZS across a range of concentrations (figure 1). For Series 1, the change in 3kPZS release rates between the baseline and 10 min periods was greater than the control for all 5d-3kPZS treatments (treatment × time ANOVA-type statistic (ATS) = 5.25; d.f. = 5.63; *p* < 0.001; all post hoc BH *p* < 0.02) but not different among 5d-3kPZS treatments (post hoc BH *p*’s > 0.17; figure 1). The same pattern held in Series 2, with release rates increasing after exposure to all 5d-3kPZS treatments (treatment × time ATS = 4.31; d.f. = 2.82; *p* = 0.006; all post hoc BH *p* < 0.03) but not differing among 5d-3kPZS treatments (post hoc BH *p*’s > 0.85; figure 1). As described above, the 5d-3kPZS treatments for Series 3 could not be compared to a negative control, but the 10 min release rates were higher than the baselines for the 10^−12^ M (Wilcoxon signed rank *p* = 0.004) and 10^−13^ M (Wilcoxon signed rank *p* = 0.01) 5d-3kPZS treatments, no different for the 10^−14^ M 5d-3kPZS treatment (Wilcoxon signed rank *p* = 0.7), and lower for the 10^−15^ M 5d−3kPZS treatment (Wilcoxon signed rank *p* = 0.002; figure 1). The increase in 3kPZS release rate after exposure to 5d-3kPZS was not different for 10^−12^ M versus 10^−13^ M (treatment × time ATS = 0.16; d.f. = 1; *p* = 0.89).

**Figure 2.**
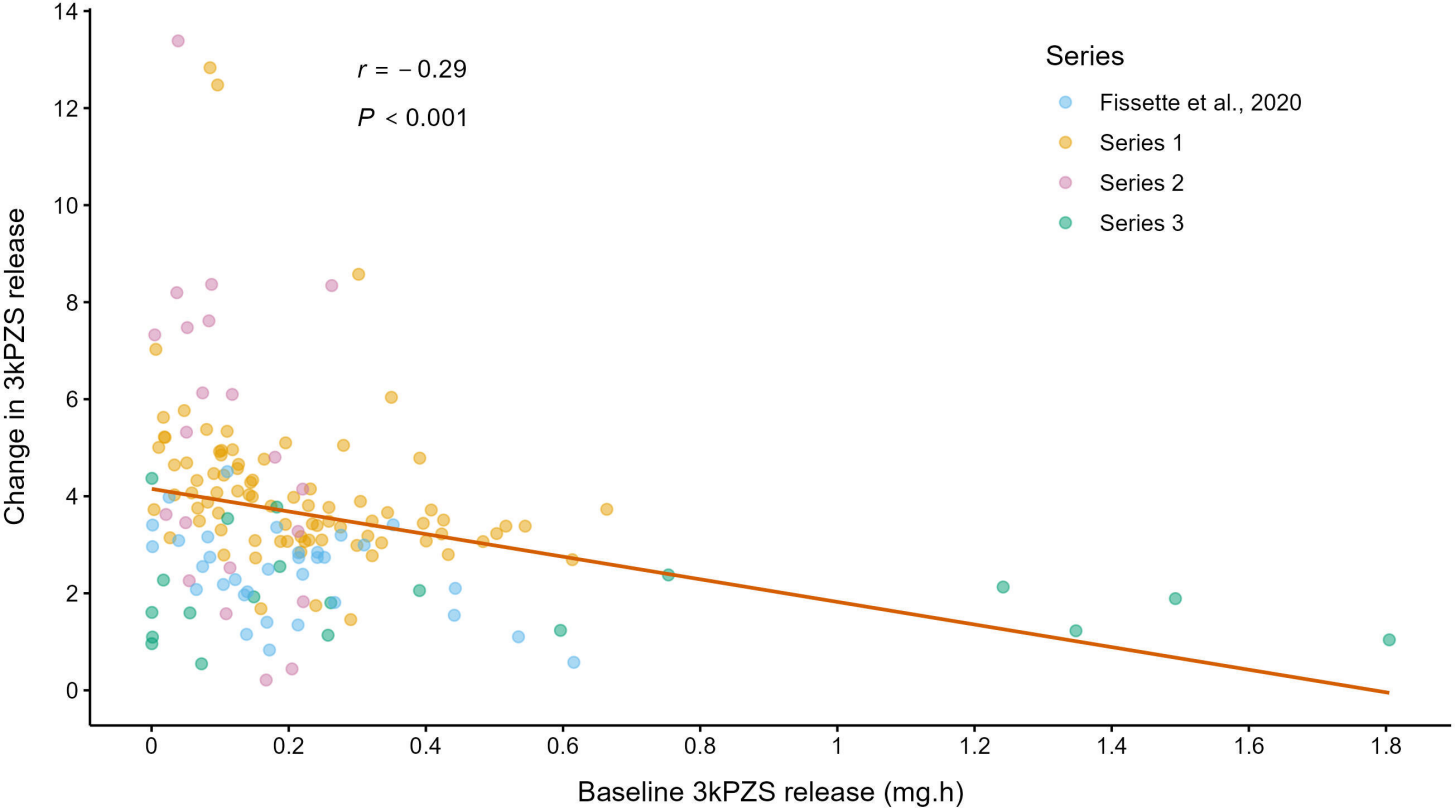
Male sea lamprey (*Petromyzon marinus*) with lower baseline signalling rates show disproportionately higher increases in pheromone release after exposure to a simulated competitor. Change in 3kPZS release after exposure to 3kPZS was calculated as the end release rate after exposure divided by the baseline release rate before exposure. Data are included for exposure treatments that evoked increased 3kPZS release in males, both in the current study (10^−7^ M to 10^−13^ M 5d-3kZPS) and Fissette *et al.* [15]. The *p*-value was generated using the correlation test described by Tu [38].

The increase in 3kPZS release rates after exposure to 5d-3kPZS was negatively correlated with the baseline 3kPZS release rate prior to 5d-3kPZS exposure (figure 2). Males with baseline release rates in the first quartile increased 3kPZS release by 325 ± 37% (*n* = 40; mean ± s.e.), whereas males with baseline release rates in the third quartile increased 3kPZS by 200 ± 23% (*n* = 40).

## 4. Discussion

Our results indicate male sea lamprey adjust their pheromone signals based on the presence of rivals but not any graded assessment of competition risk. Consistent with previous work [15], our data showed that males released the sex pheromone 3kPZS at higher rates after exposure to synthesized 3kPZS. However, exposure to 3kPZS at concentrations ranging across six orders of magnitude resulted in similar increases in 3kPZS release. In streams, variation in 3kPZS concentrations may show little correlation with competitive risk due to male variation in 3kPZS release and variable patterns of signal distribution associated with transmission through flowing water [28]. Indeed, a recent study on sea lamprey indicated a male’s location may be as important as his signalling rate in attracting females, probably due to plume dynamics that influenced the concentration of pheromone females perceive [39]. Environmental conditions shape the signals that receivers perceive via any sensory modality [29,40] and probably add noise to any relationship between competition risk and the concentration of rivals’ pheromones detected by male sea lamprey. We interpret our results in the context of sea lamprey ecology but note that laboratory studies such as ours lack features of the natural environment that can affect responses to pheromones [41].

An important caveat is that we only tested the major component of a multi-component pheromone mixture. 3kPZS is the most abundant molecule in male odorant [18] and replicates the natural pheromone mixture in attracting females to nests over long distances [23]. However, both 3kPZS and minor components influence female behaviour on or near a male’s nest [23,42]. The small active space of minor components may result from low olfactory sensitivity of females, low release rates of males or both [23]. If minor components have a similarly small active space for males, they may better reflect (compared to 3kPZS) the level of competitive risk in relation to the proximity of rivals—males may not detect minor components unless nesting very near a rival and directly competing for females with that rival. In túngara frogs, males increase calling rates after hearing rivals but only adjust this response according to the distance from which the cues originate when they perceive the ripples produced when rivals call [43]. Similarly, male sea lamprey could assess the distance to competitors using 3kPZS and minor pheromone components and countersignal at higher rates after exposure to the complete pheromone mixture versus 3kPZS alone. An incomplete understanding of the identities of minor components makes testing this hypothesis challenging.

Our data indicate a male’s baseline signalling rate influences their response to a competitor signals. Males with low 3kPZS release rates prior to 3kPZS exposure made proportionally larger increases to their 3kPZS release rates after exposure. Garcia *et al.* [44] observed a similar pattern in eavesdropping male grey tree frogs (*Hyla cinerea*), though, unlike male sea lamprey, male tree frogs signalled less in response to simulated competition if their own baseline signal was less attractive. Nevertheless, both examples indicate potential for self-assessment [30]. In sea lamprey, at least two potential mechanisms could underlie potential self-assessment: First, males with low baseline release rates could have chronically low baselines (low lifetime release rates) and alter their signals more in response to rivals. Indeed, male green frogs (*Rana clamitans*) with high-frequency (less competitive) calls adjust their signals in response to rival calls more than males with low-frequency (more competitive) calls [45]. Second, males with low baseline release rates could have temporarily low baselines (average lifetime release rate but low instantaneous release rate) and, as a result, have the additional energy or pheromone reserves to make proportionally larger changes to their signalling rates. As males in the wild will generally not smell themselves, any ‘self-assessment’ is probably based on internal physiological conditions rather than direct detection of their own signal. Multiple factors are known to influence pheromone production in sea lamprey, but additional work is needed to explain intra- and inter-male variation in 3kPZS release [25].

Sea lamprey show profound sensitivity to male pheromones [25]. Females find mates by navigating towards the sex pheromone 3kPZS at concentrations as low as 1 × 10^–14^ M [23]. In the current study, we found evidence that males seek a competitive advantage over rivals by increasing their pheromone release after exposure to 5 × 10^–13^ M or higher concentrations of 3kPZS. Though females appear to respond at a lower concentration than males, our results indicate males are keenly perceptive of changes in 3kPZS concentrations. Males in our experiments released 3kPZS to reach an average concentration of 7 × 10^–8^ M (approx. 60 ng s^–1^) immediately before responding to the addition of as little as 0.74 ng 5d-3kPZS (5 × 10^–13^ M treatment). How males detect such a seemingly minor change is unclear but could involve three potential mechanisms: First, males could perceive a change in the rate at which the 3kPZS concentration increased. We observed males respond to a 1% estimated rate of change, which is comparable to the sensitivity of some insects to rates of change in odourant concentrations [46] and intensities of other environmental stimuli (e.g. humidity) [47]. Second, males could perceive the change in pheromone component ratios. As discussed above, we exposed males to one (3kPZS) of an unknown number of male pheromone components. Therefore, the ratio of 3kPZS to minor components in the male’s holding water changed when we added 5d-3kPZS. As many animals [48], sea lamprey can discriminate some odorant mixtures based on component ratios [49,50]. Third, males might discern between 3kPZS and 5d-3kPZS based on the extra neutron on the C5 hydrogen. Evidence that animals can discriminate between isotopes is mixed [51] and there is no evidence sea lamprey respond differently to 5d 3kPZS versus 3kPZS [52]. Regardless of the mechanism, the sensitivity we observed involved males autodetecting their own pheromone in static water and may not have a major ecological function, as males in nature spawn in flowing water and are unlikely to experience consistently increasing 3kPZS concentrations.

Our study adds to a growing dataset on the internal and external factors underlying variation in sea lamprey pheromone signalling [15,20,21] and further develops this species as a model of how sexual selection shapes chemical communication [53,54].

## Data Availability

Data and R code are available from the Dryad Digital Repository [55].
